# Cerebrospinal Fluid Melanin-Concentrating Hormone (MCH) and Hypocretin-1 (HCRT-1, Orexin-A) in Alzheimer’s Disease

**DOI:** 10.1371/journal.pone.0063136

**Published:** 2013-05-07

**Authors:** Frank M. Schmidt, Juergen Kratzsch, Hermann-Josef Gertz, Mandy Tittmann, Ina Jahn, Uta-Carolin Pietsch, Udo X. Kaisers, Joachim Thiery, Ulrich Hegerl, Peter Schönknecht

**Affiliations:** 1 Department of Psychiatry and Psychotherapy, University Hospital Leipzig, Leipzig, Germany; 2 Institute of Laboratory Medicine, Clinical Chemistry and Molecular Diagnostics, University Hospital Leipzig, Leipzig, Germany; 3 Department of Anesthesiology and Intensive Care Medicine, University Hospital Leipzig, Leipzig, Germany; New York State Institute for Basic Research, United States of America

## Abstract

Ancillary to decline in cognitive abilities, patients with Alzheimer’s disease (AD) frequently suffer from behavioural and psychological symptoms of dementia (BPSD). Hypothalamic polypeptides such as melanin-concentrating hormone (MCH) and hypocretin-1 (HCRT-1, orexin-A) are promoters of sleep-wake regulation and energy homeostasis and are found to impact on cognitive performance. To investigate the role of MCH and HCRT-1 in AD, cerebrospinal fluid (CSF) levels were measured in 33 patients with AD and 33 healthy subjects (HS) using a fluorescence immunoassay (FIA). A significant main effect of diagnosis (F(1,62) = 8.490, p<0.01) on MCH levels was found between AD (93.76±13.47 pg/mL) and HS (84.65±11.40 pg/mL). MCH correlated with T-tau (r = 0.47; p<0.01) and P-tau (r = 0.404; p<0.05) in the AD but not in the HS. CSF-MCH correlated negatively with MMSE scores in the AD (r = −0.362, p<0.05) and was increased in more severely affected patients (MMSE_≤20_) compared to HS (p<0.001) and BPSD-positive patients compared to HS (p<0.05). In CSF-HCRT-1, a significant main effect of sex (F(1,31) = 4.400, p<0.05) with elevated levels in females (90.93±17.37 pg/mL vs. 82.73±15.39 pg/mL) was found whereas diagnosis and the sex*diagnosis interaction were not significant. Elevated levels of MCH in patients suffering from AD and correlation with Tau and severity of cognitive impairment point towards an impact of MCH in AD. Gender differences of CSF-HCRT-1 controversially portend a previously reported gender dependence of HCRT-1-regulation. Histochemical and actigraphic explorations are warranted to further elucidate alterations of hypothalamic transmitter regulation in AD.

## Introduction

In Alzheimer’s disease (AD) which is predominately characterized by the decline of cognitive abilities in memory, abstraction, orientation and language, cognition-related behavioral and psychological symptoms of dementia (BPSD) frequently occur. These include sleep-wake disturbances with daytime sleepiness and napping, nightly awakening, reduced REM-sleep and increased REM-onset latency, cyclic agitation and deregulation of appetite and weight [1], [2].

The complementary acting transmitters melanin-concentrating hormone (MCH) and hypocretin-1 (HCRT-1; syn. orexin-A) are synthesized in the hypothalamus, a subcortical region where senile plaques and neurofibrillary tangles as ‘hallmarks of dementia’ are to be found [3], [4], [5]. Experimental studies demonstrate that receptors for both peptides are found in areas not only involved in the regulation of wakefulness, circadian rhythmicity and energy homeostasis but also regarded affected in AD, such as the cerebral cortex, cholinergic neurons of the basal forebrain, amygdala, and the brainstem [6], [7], [8], [9], [10], [11]. Animal studies have shown that the activation of MCH neurons leads to improved learning and memory performance (for references see [8]), increased REM sleep [12], [13] increased food and water intake [14] and has anxiogenic effects in mice [15]. Reduction of MCH or blockage of MCH-receptors leads to improved social recognition in rats [16], hyperactivity, leanness, hypermetabolism in mice [17], and has antidepressant effects in animals (for references see [18]).

The infusion of HCRT-1 improves accuracy in demanding attentional tasks in rats [19] and the excitement of neurons of the basal forebrain [20]. In HCRT-deficient narcoleptic patients, the dysregulation of sleep-wake control is featured by worsening in cognitive attentive processing as well as performance in information gathering and memory tasks [21], [22], [23], [24].

While to date no data on MCH levels in AD exist, literature concerning HCRT-1 is heterogenous: whereas immunoreactive neurons in postmortem hypothalami and ventricular cerebrospinal fluid (CSF)-HCRT-1 levels in AD patients were found to be reduced [25], lumbar CSF levels in vivo were unaltered [26], [27], [28]. A regulation of amyloid-beta (Aβ42) by HCRT has been concluded since the infusion of HCRT-1 in animals lead to increased levels of Aβ42 in the brain interstitial fluid. In contrast the infusion of an HCRT-1-antagonist led to a reduction in Aβ42-plaque deposition as shown with microdialysis in mice [29]. Only in AD but not healthy subjects (HS), daily amplitudes of HCRT-1 and Aβ42 correlated [30] whereas levels were not found to correlate in neither AD nor HS [26]. Furthermore in AD, CSF-HCRT-1 levels negatively correlated with the number of daily naps and the total amount of daily nap time [31]. A reduction of HCRT-1-containing neurons [32] and HCRT-1-mediated efflux of acetylcholine in the medial septum [33] with age has been furthermore demonstrated in rats.

The aim of this study was to investigate the CSF levels of MCH and HCRT-1 in patients with AD and HS and the potentially causal relationship between these polypeptides and CSF levels of the AD marker total Tau (T-tau), hyperphosphorylated tau (P-tau) and Aβ42, cognitive performance and behavioral symptoms in AD.

## Methods

### Subjects

This study was approved by Leipzig University and Saxony Medical Ethics Committee. Patients or, when appointed, proxy provided written informed consent to the appropriation in this clinical study.

33 patients with mild to severe AD and 33 subjects without any psychiatric or neurological disorder (HS) were consecutively recruited to participate in the study. Characteristics of patients and HS such as age, gender, psychotropic medication as well as performances in cognitive tests are depicted in tables 1 and 2. All patients and HS were admitted to the Department of Psychiatry of the University Hospital Leipzig as inpatients. Patients or HS showing any indication of limitation to provide full consent and lacking a proxy were excluded from the study. Patients with AD were admitted in order to clinically explore and diagnose cognitive deficits registered in ambulatory setting. Patients were diagnosed with AD under supervision of a senior specialist in geriatric psychiatry according to Dubois criteria [34]. Diagnostics included history, clinical investigation, neuropsychological testing with the neuropsychological test battery of the Consortium to Establish a Registry for Alzheimer’s Disease (CERAD; [35]), Wechsler Memory Scale-Revision (WMS-R; [36]), clock drawing test [37], and Trail Making Test (TMT; [38]), a MRI head-scan, genotyping of ApoE4 and determination of Aβ42, T-tau and P-tau in the CSF. Patients were only included when no further neurological disturbances were diagnosed by clinical and laboratory investigations. BPSD was categorized into sleep disorder and irritability. Sleep disorder contained sleep onset latency of more than two hours or fragmented sleep with 1 or more periods of awakening for more than half an hour during the in the night before investigation. Irritability included verbal aggression, including curse and abusive formal language recorded by physicians or nursing staff.

**Table 1:** Subject characteristics.

	AD	HS
**N**	33	33
**Male/female**	11/22	19/14
**Age in years (±SD)**	73.76 (±8.07)	52.03 (±17.24)***
**MMSE (±SD)**	19.76 (±6.54)	29.92 (±0.29)***
**BMI (±SD)**	24.97 (±4.61)	27.44 (±5.15)
**CSF-t-Tau in pg/ml (±SD)**	449.5 (±245.0)	133.8 (±48.5)***
**CSF-pTau in pg/ml (±SD)**	79.2 (±38.8)	32.6 (±12.0)***
**CSF-Amyloid-β-1-42 in pg/ml (±SD)**	633.8 (±290.2)	1157.3 (±260.6)***
**Psychotropic medication n/y**	22/11	33/0
Galantamine	3	―
Rivastigmine	4	―
Memantine	3	―
Venlafaxine	2	―
Citalopram	1	―
Risperidone	1	―

*** p<0.001, N = Number, AD = Alzheimer’s Disease, HS = Healthy subjects, N = Number, SD = Standard Deviation, BMI = Body Mass Index.

**Table 2:** Clinical characteristics of patients with AD.

	Total AD	Male AD	Female AD
**N**	33	11	22
**Age (±SD)**	73.76 (±8.07)	73.36 (±7.02)	73.95 (±8.71)
**BMI (±SD)**	24.97 (±4.61)	24.78 (±5.14)	24.05 (±4.47)
**MWT-A (±SD)**	24.46 (±9.25)	29.38 (±4.47)	22.28 (±10.06)
**Education in years (±SD)**	9.20 (±1.54)	10.1 (±1.79)	8.75 (±1.21)*
**MMSE (±SD)**	19.76 (±6.54)	19.82 (±5.86)	19.73 (±6.99)
**TMT-A (±SD)**	123.83 (±74.53)	107.00 (±82.07)	125.09 (±75.57)
**TMT-B (±SD)**	251.96 (±89.71)	234.13 (±153.41)	206.42 (±111.63)
**Clock drawing test (±SD)**	3.61 (±1.65)	3.44 (±1.94)	3.68 (±1.55)
**WMS-Logical Memory-I (±SD)**	6.03 (±5.38)	8.44 (±6.75)	5.05 (±4.52)
**WMS-Logical Memory-D (±SD)**	2.87 (±4.81)	3.89 (±5.62)	2.45 (±4.51)
**Verbal fluency (±SD)**	9.81 (±4.88)	9.89 (±5.25)	9.77 (±4.84)
**MBNT (±SD)**	10.87 (±3.49)	13.44 (±1.42)	9.82 (±3.56)**
**Wordlist learning (±SD)**	9.83 (±5.09)	10.89 (±4.70)	9.38 (±5.29)
**Wordlist recall (±SD)**	4.20 (±13.47)	2.11 (±1.83)	5.10 (±16.09)
**Constructional praxis learning (±SD)**	8.32 (±2.51)	9.11 (±1.97)	8.00 (±2.67)
**Constructional praxis recall (±SD)**	1.71 (±2.037)	2.89 (±2.21)	1.23 (±1.79)
**Apo E4 carriers in %**	65.4	77.8	58.8

* p<0.05,

** p<0.01, N: Number, SD: Standard Deviation, BMI: Body Mass Index, MWT-A: Mehrfachwahl-Wortschatz-Test A, MMSE: Mini Mental State Examination, TMT: Trail Making Test, Scale Logical Memory- Delayed recall trial, MBNT: Modified Boston, Naming Test, WMS-Logical Memory- I: Wechsler Memory Scale-Logical Memory-Immediate recall trial, WMS-Logical Memory- D: Wechsler Memory Scale-Logical Memory- Delayed recall trial.

HS were recruited from the Clinic of Anesthesiology and Intensive Care Medicine, University Hospital Leipzig and Helios Hospital Borna, prior to elective abdominal or orthopedic surgery. HS showing any signs of neurological or psychiatric disorder in clinical and laboratory investigation or with the medication of psychoactive drugs within the last 30 days were excluded from the study. In order to further survey exclusion criteria, the Structured Clinical Interview Axis I Disorder (SCID; [39]), the Hamilton Depression Rating Scale (HAMD-17; [40]) and the Mini Mental Status Examination (MMSE; [41]) were performed when applicable.

### Processing of Neuropeptides

In order to minimize known diurnal variation as a confounding factor which is found for HCRT-1 and assumed for MCH [30], [42], lumbar puncture was performed in a time slot between 00:30 pm and 01:30 pm. Following, samples were immediately aliquoted in non-absorbing polypropylen-tubes of 300 µl. Probes were shock-frozen in fluid N2 and stored in freezers at −80°C until further measurements. For the measurement of MCH we used a fluorescence immunoassay (FIA) with a linear measuring range between 35.0 and 808 pg/mL (Phoenix Pharmaceuticals, Burlingame, US). For the measurement of HCRT-1 we used a fluorescence immunoassay (FIA) with a linear measuring range between 15.2 and 366 pg/mL (Phoenix Pharmaceuticals, Burlingame, US).

### Statistical Analysis

The IBM Statistical Package for the Social Sciences (SPSS) program version 20.0 for Windows was used for all statistical analyses. The significance level was set at p<0.05 for all statistical analyses. Univariate ANOVA with post-hoc Bonferroni testing for multiple comparisons was performed for analysis of MCH and HCRT-1 between AD and HS, between sexes, between medicated and unmedicated patients and HS, for patients showing any of the BPSD stated above (BPSD+) and showing no BPSD (BPSD−), in patients with mild severities of AD (MMSE_≥21_) and moderate to severe severities (MMSE_≤20_), [43], as well as for detection of interactions between groups and gender. T-test was performed for differences of MCH and HCRT-1 in ApoE4 carriers vs non-carriers, as well as mean differences in scores in cognitive tests and educational level between male and female AD. For bivariate correlation of HCRT-1 and MCH levels and cognitive testing, Pearson’s correlation was performed; for BMI and age, two- tailed analyses of coefficient was performed using Spearman Rho. To assess the accuracy rates of MCH, receiver operating characteristic (ROC) analyses resulting in area under the curve values (AUC) was performed. Sensitivity and specificity was computed for different cut-off scores. The Youden index was used to select optimal cut-off scores.

## Results

**Figure 1:**
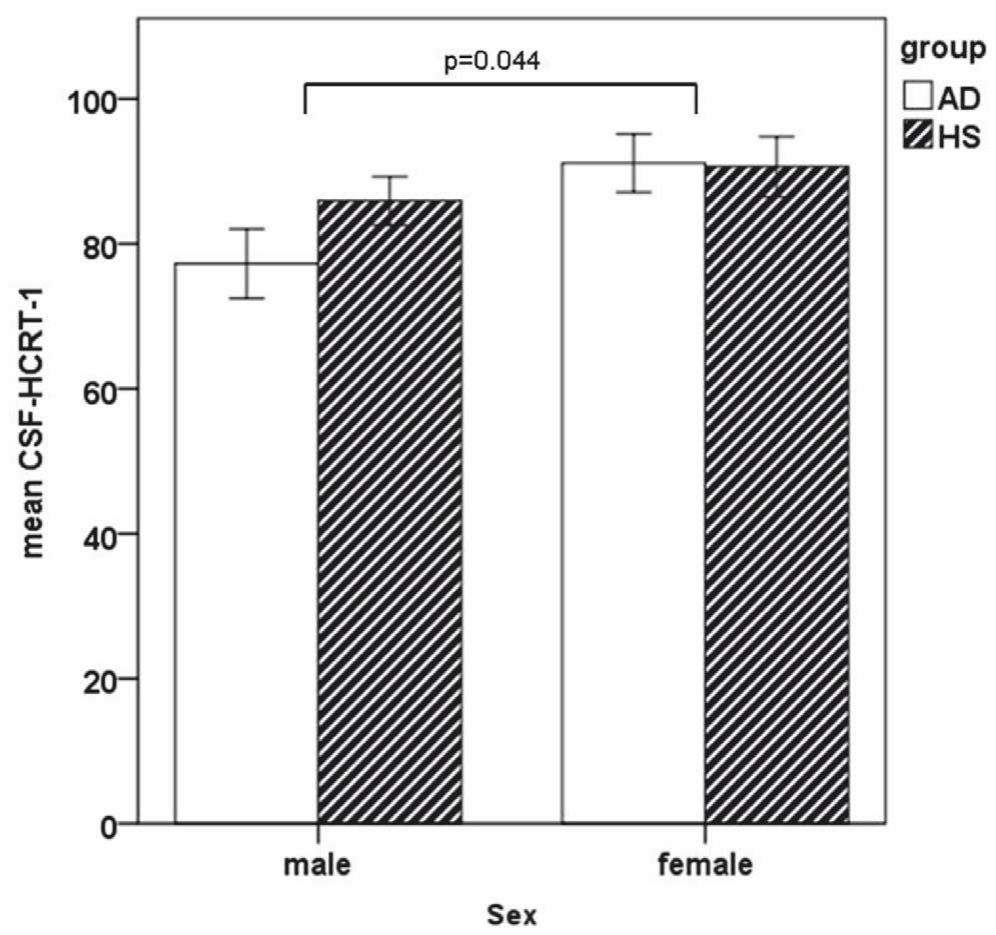
Mean value of CSF-MCH was elevated in AD when compared to HS.

The distributions of CSF-MCH and CSF-HCRT-1 within the groups are depicted in table 3. Regarding MCH, the inferential statistical analyses showed a main effect of diagnosis (F(1,62) = 8.490, p<0.01) (see figure 1), whereas age (F(38,66) = 1.338, p = 0.237), sex (F(1,62) = 0.424, p = 0.517) and diagnosis*age interaction (F(4,66) = 0.957, p = 0.450) and diagnosis*sex interaction (F(1,62) = 1,175, p = 0.283) were not significant. MCH correlated with T-tau (r = 0.47, p<0.01) and P-tau (r = 0.404, p<0.05) in the AD but not in the HS. MCH and Aβ42 were found to weakly correlate in the total group (r = −0.264, p<0.05) but not in separate groups. CSF-MCH levels showed a negative correlation with MMSE scores in AD (r = −0.362, p<0.05) but not in the HS. Of the further cognitive tests performed, negative correlation was found with scores in the TMT-B (r = −0.466; p<0.05). CSF-MCH levels differed between AD with MMSE_≤20_ (n = 15), AD with MMSE_≥21_ (n = 18) and HS, (F(2,62) = 6.997, p<0.01) with post-hoc analyses showing higher levels in AD with MMSE_≤20_ compared to HS (p<0.001). CSF-MCH differed between BPSD+ (n = 14), BPSD− (n = 19) and HS (F(2,63) = 4.623, p<0.05) with post-hoc analyses showing higher levels in BPSD+ compared to HS (p<0.05). CSF-MCH levels also differed between medicated AD (n = 11), drug-naïve AD (n = 22) and HS (F(2,63) = 4.664, p = 0.05), with post-hoc analyses showing higher levels in medicated AD compared to HS (p<0.05). For the differentiation between AD and HS, ROC analyses for controlled values resulted in AUC = 0.713. With a Youden index of 0.39, sensitivity was 67% and specificity 73% for a cut-off value of 90.15 pg/mL.

**Table 3:** Levels of CSF-MCH and -HCRT-1 in patients with AD and HS.

	CSF-MCH in pg/mL		CSF-HCRT-1 in pg/mL	
	AD	HS	AD	HS
**Mean (±SD)**	93.76 (±13.47)	84.65 (±11.40)**	86.50 (±18.82)	87.91 (±14.95)
**Female (±SD)**	94.22 (±14.55)	81.46 (±12.07)**	91.12 (±18.75)	90.62 (±15.61)
**Male (±SD)**	92.84 (±11.60)	87.19 (±10.85)a	77.26 (±15.94)	85.91 (±14.53)*
**AD MMSE≥21 (±SD)**	89.52 (±13.53)	84.65 (±11.40)a	84.21 (±19.69)	87.91 (±14.95)
**AD MMSE≤20 (±SD)**	98.84 (±11.90)	“ ***	91.08 (±16.89)	“
**AD BPSD– (±SD)**	92.42 (±13.30)	“	88.00 (±20.37)	“
**AD BPSD+ (±SD)**	95.58 (±13.85)	“ *	84.69 (±17.31)	“
**AD Med– (±SD)**	92.58 (±15.54)	“	86.96 (±21.43)	“
**AD Med+ (±SD)**	96.12 (±15.54)	84.65 (±11.40)*	85.88 (±15.34)	87.91 (±14.95)

* p<0.05;

** p<0.01;

*** p<0.001, AD = Alzheimer’s Disease, HS = Healthy subjects, N = Number, SD = Standard Deviation, AD Med−/+ = AD patients without/with psychotropic medication, MMSE≥21/≤20: Scores in the Mini Mental State Examination higher or equal 21 points or less or equal 20 points, BPSD+/−: presence or absence of behavioral and psychiatric symptoms of dementia.

**Figure 2:**
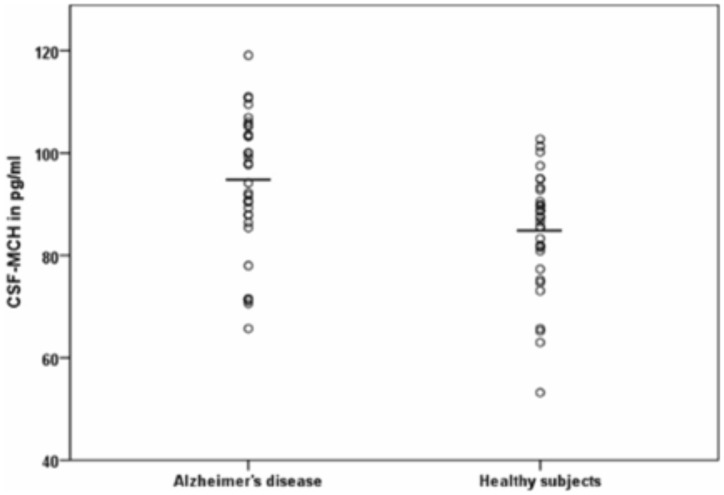
In HCRT-1, mean differences in gender were found as main effect with reduced levels in males.

Regarding CSF-HCRT-1, the inferential statistical analyses showed a main effect of sex (F(1,62) = 4.863, p<0.05), whereas diagnosis (F(1,62) = 0.937, p = 0.337), age (F(38,66) = 1.408, p = 0.199), the sex*group (F1,62) = 1.179, p = 0.282) and age*group (F4,66) = 0.858, p = 0.504) interaction did not reach significance (see figure 2). CSF-HCRT-1 did not correlate with the MMSE in the two groups. Among the other cognitive tests performed, significance in the AD was reached with the TMT-A (−0.412, p<0.05). CSF-HCRT-1 did neither differ in means between groups of medicated AD, drug-naïve AD and HS nor between groups of BPSD+, BPSD− and HS. HCRT-1 did not correlate significantly with T-tau, P-Tau or Aβ42. CSF-MCH and CSF-HCRT-1 correlated within the AD group, r = 0.382, p<0.01, but not in the HS. Levels of CSF-MCH and CSF-HCRT-1 did not correlate with age, education levels in school years, MWT-A or BMI, and no differences in means of CSF-MCH or CSF-HCRT-1 depending on the ApoE4 genotype were found.

## Discussion

In the present study, we investigated CSF levels of the hypothalamic polypeptides MCH and HCRT-1 in 33 patients with AD and 33 HS. This, to the best of the authors’ knowledge, first investigation of MCH in AD revealed elevated means in the CSF when compared to HS. To differentiate AD from HS, a moderate sensitivity and specificity was reached with the respective optimal cut-off. Furthermore, levels of CSF-MCH were found to correlate with T-tau and P-tau only in the AD and to be severity- and BPSD-related. An association between CSF levels of MCH and HCRT-1 was found only in AD patients, whereas means of HCRT-1 were not found to differ significantly between AD and HS. Notably, CSF-HCRT-1 levels showed sex- dependence with elevations in females when compared to males.

A couple of findings suggest the regulation of MCH to be altered in AD. Correlations between MCH and Tau, but not for Aβ42, as established marker for the neuropathic formation of neurofibrillary tangles only in AD portend a connection between the parameter. The formation of neurofibrillary tangles in the hypothalamus in AD [5] with its expression of elevated levels of Tau may have impact on the activity of MCH-ergic neurons leading to a, potentially temporary, hypersecretion of MCH. MCH was further linked to severity of the disorder in general since only MMSE and TMT were found to correlate. In line with the regulatory function on sleep-wake regulation MCH was connected to the occurrence of BPSD. Although means of MCH did not show to differ by more than about 10 percent and although levels of MCH between AD and HS were found to overlap in a large proportion of participants which makes it rather useless as diagnostic or differential tool in AD, the finding of elevated means in a neurodegenerative disease is noteworthy. According to our findings and the impact of other chronobiological agents such as melatonin on neurodegenerative symptomatology [44], an explanatory approach may be driven here. Besides the neurodegenerative influence of the formation of neurofibrillary tangles, changes in levels of MCH may depict changes in the MCH-neuron regulation: MCH-receptor (MCH-R) sites are pronounced found in the hippocampus and cortex, vulnerable areas for degeneration in AD. Reduced stimulation or presence of MCH-R leads to a reduced negative feedback and increased high voltage-activated calcium channels in MCH neurons in the LH [45], leading consecutively to higher release of MCH. Supporting a modification of MCH-R in AD, MCH-R deficient mice showed worse cognitive performance and reduced N-methyl D-aspartate (NMDA) receptors in the hippocampus [46]. The positive effects of MCH on performance in learning and memory [47], the potentiation of hippocampal synaptic transmission by infusion of MCH [48] may be affected in AD due to reduced MCH-R availability. By primarily stabilizing REM-sleep phases [6], [12] an indirect effect of MCH on memory abilities has been shown [8]. Patients with AD lack the impact of (REM−) sleep on memory consolidating and synaptic plasticity [49], [50] which may contribute to cognitive impairment [51].

It may be considered if elevated CSF-MCH levels are caused by a temporal release of polypeptides subsequent to cellular apoptosis or necrosis in the LH. However, no short or long-time increase but only a reduction of transmitter levels are found [52] and no correlation between cell loss and CSF-levels could be found for HCRT-1 in AD [25].

In contrast to a previous report [25], we did not find differences in CSF-HCRT-1 between the two groups. Whereas patients in our study suffered from mild to severe AD, samples in the aforementioned were drawn from post-mortal brains. Gerashchenko and colleagues [52] demonstrated that the loss of a high proportion of HCRT-containing neurons leads to significant alterations of CSF-levels, possibly a state of degeneration patients in this study did not yet suffer from.

When performing subgroup analysis, our data showed increased CSF-HCRT-1 in females compared to males. This finding supports a previous report in which female AD patients showed increased levels in CSF-HCRT-1 compared to male AD patients, healthy females and females with DLB [26]. Thereupon, a determining influence of gender in the regulation of CSF-HCRT-1 was postulated. Findings indicating higher incidence of AD in females [53], worse cognitive performance in female mice in a transgene mice model of dementia [54], pronounced impairment of sleep in females [55], the modulation of HCRT-receptor-1 by gonadal steroids [56] and a sexually dimorphic expression of HCRT-1 [57] make further investigation of a gender dependence of HCRT-1 regulation in AD warranted. Contrary to the prior investigations stated in the introduction, we did neither find correlations between HCRT-1 and Tau nor Aβ42 and may therefore not support a link between established pathological cascades and HCRT-1.

When interpreting the results and the use of cut-off scores in clinical practice, several limitations have to be considered. A third of the patients with AD received psychotropic medication that may have had influence on MCH and HCRT-1 neuronal activity. Furthermore, groups differed in age and gender distributions. Though no impact of age and gender in the inferential statistics and no correlations between age and polypeptide levels were found in this nor previous reports [58], [25], these factors as well as the psychotropic medication cannot fully be ruled out to account for the group differences of MCH levels. Since biomarkers in AD have been demonstrated to show characteristic time course [59], further studies have to control for duration of illness. Concerning the objection of BPSD, data from medical records as used here are potentially not of enough accuracy and should be added by established rating scales assessing appetite sleep and behavioral qualities. Moreover, the binary differentiation between presence or absence of any BPSD and no further itemization is potentially of impreciseness.

This report on MCH in patients with AD adds to preclinical studies and may allow further research on the role of the hypothalamus in pathology and occurrence of behavioral disturbances in AD. Future investigations, including repeated MCH/HCRT-1 level measurements in combination with sleep-wake-actigraphy and assessment of sleep by questionnaires, histochemical determinations of MCH in animal and post-mortem tissues are warranted to understand the hypothalamic impact in AD in greater detail.
