# Correction: Cerebrospinal Fluid Melanin-Concentrating Hormone (MCH) and Hypocretin-1 (HCRT-1, Orexin-A) in Alzheimer’s Disease

**DOI:** 10.1371/annotation/7a79c40c-c1c2-41eb-a894-614c12e8c056

**Published:** 2013-06-28

**Authors:** Frank M. Schmidt, Juergen Kratzsch, Hermann-Josef Gertz, Mandy Tittmann, Ina Jahn, Uta-Carolin Pietsch, Udo X. Kaisers, Joachim Thiery, Ulrich Hegerl, Peter Schönknecht

Figures 1 and 2 were incorrectly switched. 

The figure labeled as Figure 1 belongs with the title of Figure 2 and the figure labeled as Figure 2 belongs with the title of Figure 1. The titles themselves are in correct order. 

